# Molecular Interactions Between Innate and Adaptive Immune Cells in Chronic Lymphocytic Leukemia and Their Therapeutic Implications

**DOI:** 10.3389/fimmu.2018.02720

**Published:** 2018-11-26

**Authors:** Muhammad Haseeb, Muhammad Ayaz Anwar, Sangdun Choi

**Affiliations:** Department of Molecular Science and Technology, Ajou University, Suwon, South Korea

**Keywords:** B cell, chronic lymphocytic leukemia, crosstalk, dendritic cell, leukemia therapy, macrophage, T cell

## Abstract

Innate immunity constitutes the first line of host defense against various anomalies in humans, and it also guides the adaptive immune response. The function of innate immune components and adaptive immune components are interlinked in hematological malignancies including chronic lymphocytic leukemia (CLL), and molecular interactions between innate and adaptive immune components are crucial for the development, progression and the therapeutic outcome of CLL. In this leukemia, genetic mutations in B cells and B cell receptors (BCR) are key driving factors along with evasion of cytotoxic T lymphocytes and promotion of regulatory T cells. Similarly, the release of various cytokines from CLL cells triggers the protumor phenotype in macrophages that further edges the CLL cells. Moreover, under the influence of various cytokines, dendritic cells are unable to mature and trigger T cell mediated antitumor response. The phenotypes of these cells are ultimately controlled by respective signaling pathways, the most notables are BCR, Wnt, Notch, and NF-κB, and their activation affects the cytokine profile that controls the pathogenesis of CLL, and challenge its treatment. There are several novel substances for CLL under clinical development, including kinase inhibitors, antibodies, and immune-modulators that offer new hopes. DC-based vaccines and CAR T cell therapy are promising tools; however, further studies are required to precisely dissect the molecular interactions among various molecular entities. In this review, we systematically discuss the involvement, common targets and therapeutic interventions of various cells for the better understanding and therapy of CLL.

## Introduction

Chronic lymphocytic leukemia (CLL) is the most common leukemia in the Western world, with ~4.5 cases per 100,000 individuals reported annually (1). The development and progression of CLL are accompanied by several genetic abnormalities and disorders, and CLL is characterized by the gradual accumulation of maturing-looking clonally expanded CD5^+^ B lymphocytes in peripheral lymphoid organs, secondary lymphoid organs, and bone marrow. There are various signals that induce proliferation and accumulation that lead to survival of malignant cells (2). Abnormalities in the development of B cells cause CLL, immune deficiencies, malignancies, and allergies. Various mutations in hematopoietic cells and immune deficiencies are recognized in CLL, and novel studies are being designed to gain a deeper understanding of these associations. Moreover, personalized forms of treatment are being developed to treat CLL (3).

CLL cells are largely derived from the continuum of maturation states observed in normal developmental stages when compared to normal B cells. Epigenetic maturation in CLL is associated with an indolent gene expression pattern and increasingly favorable clinical outcomes (4). In addition, previously reported tumor-specific methylation events are normally present in non-malignant B cells. Moreover, a potential pathogenic role has been identified for dysregulated transcription factors in CLL, including the induction of signaling by nuclear factor of activated T cells (NFAT) and early growth response (EGR) proteins, resulting in diminished early B cell factor (EBF) and AP-1 programming compared to that in the normal B cell epigenetic program.

**Figure 1:**
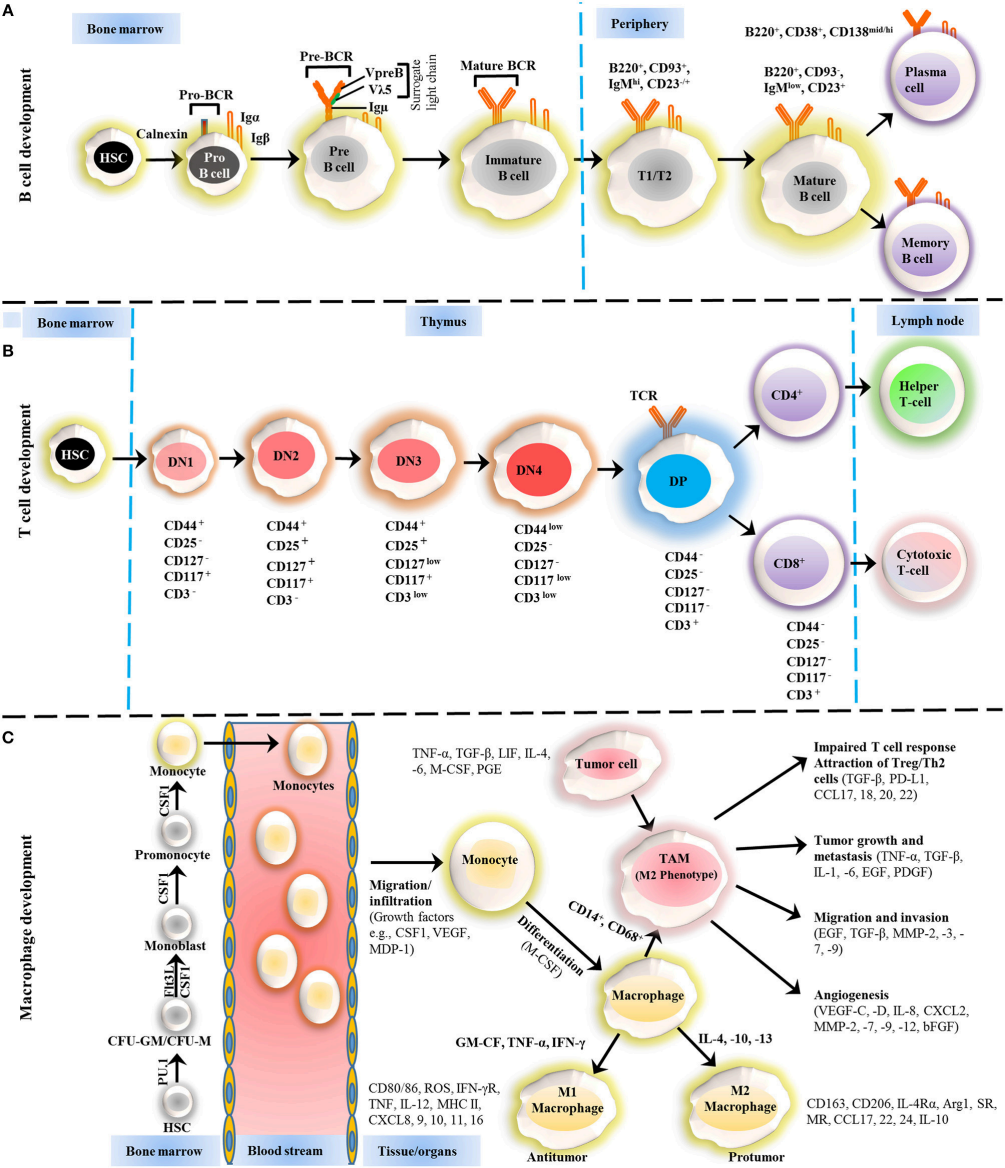
Development of B cells, T cells and macrophages. **(A)** The development of B cells occurs in the bone marrow and peripheral lymphoid tissues. Development progresses from hematopoietic precursor cells (HSCs) and proceeds through several stages, such as a pro-B cell, pre-B cell, and immature B cell. During differentiation, the pre-B-cell receptor (pre-BCR) is generated following immunoglobulin locus rearrangements and is expressed on the cell surface. This pre-BCR (consisting of the surrogate light chain [VpreB or Vλ5] and an Igμ heavy chain) undergoes further rearrangements of the light and heavy-chain genes to form a mature BCR that can bind to the antigen. A selection process occurs at this immature B cell stage that prevents self-reactive cells from developing further. This stage is accompanied by both clonal selection and receptor editing. Those cells that successfully pass through this checkpoint (named transitional B cells) leave the BM and acquire their mature form as mature follicular B cells. **(B)** T cells development starts from HSCs in bone marrow and progresses to the thymus, where it passes through a series of developmental stages that can be recognized based on the expression of different cell surface markers. In the beginning of development, the expression of co-receptors CD4 and CD8 are absent and called double negative (DN) cells. The DN cells (DN1, DN2, DN3, and DN4) are further sub-divided by the expression of CD117, CD44, CD25, CD127, and CD3 markers. Further differentiation takes place by the up-regulation of CD4 and CD8 expression, therefore, names as double positive (DP) cells. The negative selection against self-antigen occurs in the thymus (medulla), where antigens are presented to them by dendritic cells and macrophages. T cells with stronger affinity then eliminated and the remaining T cells downregulate either co-receptor CD4 or CD8 and give rise to naïve cells stay in thymus and periphery. **(C)** The macrophage development and maturation also take place in bone marrow and tissues. From HSC, myeloid colony forming units are derived in bone marrow, and further grow into monocytes under colony-stimulating factor 1 (CSF1) through various highly organized stages. These monocytes can give rise to common DC progenitor cells that can transform into blood monocytes and, upon homing to various tissues except brain and skin macrophages, tissue macrophages. During most of the developmental stages, various factors influence the macrophage lineage development, however, CSF1 is likely imparted the highest influence. Finally, to address the inflammation, monocytes are recruited to tissues and restricted to specific phenotypes, M1, M2, and tumor-associated macrophages (TAM) depending upon inflammatory milieu. The development of TAM could also be influenced by tumor cells and tissue-resident macrophages. On the other hand, some other factors released by TAMs suppress the local immune response by either directly suppressing T cell responses or recruiting Treg cells. *Arg1*, arginase 1; *bFGF*, basic fibroblast growth factor; *CCL*, chemokine (C-C motif) ligand; *CD*, cluster of differentiation; *CSF1* colony-stimulating factor-1; *CXCL*, chemokine (C-X-C motif) ligand; *FLT3*, FMS–like tyrosine kinase receptor 3; *IFN*, interferon; *Ig*, Immunoglobulin; *IL*, interleukin; *LIF*, leukocyte inhibitory factor; *MDP*, macrophage-derived proteoglycan; *MHC*, major histocompatibility complex; *MMP*, matrix metalloproteases; *MR*, Mineralocorticoid receptor; *PD-L*, Programmed death-ligand; *PGE*, prostaglandin; *SR*, scavenger receptor; *TAM*, tumor-associated macrophage; *TGF*, transforming growth factor; *TNF*, tumor necrosis factor; *VEGF*, vascular endothelial growth factor.

The immune system response can be divided into two phases; (a) innate immunity that arise from myeloid lineage cells and mature into monocytes, macrophages, erythrocytes, platelets, and granulocytes, provide the first line of defense, (b) and the adaptive immune system arises from lymphoid progenitor cells and give rise to natural killer (NK) cells, B cells, and T cells, and provides the second line of defense against pathogens and other abnormalities (Figure 1) (5, 6). These myeloid and lymphoid cells affect the progression of CLL in an independent and collaborative manner. The CLL microenvironment is populated by macrophages, and the transfer of antigens is dependent on the contact between B cells and macrophages (7). Other than antigen transfer, the influence of various cell surface receptors, cytokine secretion, and immune suppression are frequently being interconnected among these cells. Therefore, in this review, we will address the recent advances in linking these blood cells and the therapeutic approaches to counter blood malignancies, particularly CLL.

## Adaptive immune system in CLL: lymphocytes involvement in CLL pathogenesis

The adaptive immune response is mainly carried out by B cells, T cells NK cells, where, B cells are the major player in various blood-related abnormalities. For the development and maturation of B cells, the B cell receptor (BCR) is the critical mediator of the proliferation and survival of mature B cells and other precursor tumor cells, as well as, BCR mutational status, is highly correlated with the pathology of disease (8). Moreover, mutations in the receptors are thought to play pivotal roles in CLL etiopathogenesis, as 20% of CLL in unrelated patients involves the display of extremely similar and sometimes even identical antigen receptors. BCR signaling is also critical for CLL cell trafficking, interaction with stromal microenvironment, impaired CLL response, and low expression of the BCR correlates with reduced induction of protein tyrosine kinase activity.

CD5^+^ B cells are the primary cells that give rise to CLL, although a few reports implicate T cells as well. BCR signaling is the most important feature and a diagnostic marker of CLL that can drive CLL progression in an antigen-independent manner (9). Along with BCR, several genetic modifications are also frequently reported to be causative agents of CLL, and such genetic abnormalities act in synergy with various cells types, such as stromal cells, T cells, and nurse-like cells (NLC) in the lymph nodes (10). Numerous defects affecting downstream signaling proteins in the BCR pathway and mutations in the interleukin 7 (IL-7) receptor are not direct causes of CLL; however, such factors predispose cells to develop into CLL cells, and they also influence the development of B and T cell malignancies and severe immunodeficiencies (11, 12). Moreover, B cell development in humans is heavily reliant on Bruton's tyrosine kinase (BTK) signaling activity, as this pathway regulates the activity of various transcription factors, and any errors in this pathway severely impact and even block B cell maturation (Table 1) (43).

**Table 1:** B cell lymphomasm and mechanisms of pathogenesis.

**Lymphoma**	**Characteristics**	**Suggested cellular origins**	**Chromosomal translocations**	**Tumor suppressor gene mutations**
B-CLL	B cell leukemia with the expression of antigen CD5 in bone marrow cells and peripheral blood. The prognosis is worse when the normal V region gene is expressed.	MZ MB Naive B cell	NA	*ATM* [30%], *TP53* [15%] (13, 14)
Mantle cell lymphoma	Begins in the mantle zone of follicles, expresses CD5, and shows anomalies in the expression of cyclin D1. Almost all cases are linked to changes in BCL1-IgH.	CD5^+^ Mantle zone	*CCND1-IgH* [95%] (15)	*ATM* [40%] (16)
Lymphocyte-predominant Hodgkin's lymphoma	Shows a specific B cell phenotype in tissues. Grows in conjunction with follicular dendritic and T helper cells.	GC	*BCL6*-various [48%] (17)	NA
Classical Hodgkin's lymphoma	Large tumor cells. Reed-Sternberg and Hodgkin's cells account for >1% of tumor cells.	Abnormal GC	NA	*IKBA* [10–20%] (18), *IKBE* [10%] (19), *CD95* [<10%] (20)
Multiple myelomas	Plasma cells proliferate in the bone marrow.	Plasma cells	*CCND1-IgH* [15–20%] (21), *FGFR3-IgH* [10%] (22), *MAF-IgH* [5–10%] (23)	*CD95* [10%] (24)
Lymphoplasmacytic lymphoma	This cancer involves bone marrow, spleens, and lymph nodes and is composed of small B cells. Patients' sera exhibit monoclonal protein IgM.	Post GC	*PAX5-IgH* [50%] (25)	NA
Primary effusion lymphoma	Mostly present in AIDS or organ transplant patients. Such type of lymphoma found in cavities, pleura, and pericardium.	Post GC	NA	NA
Post-transplant lymphoma	Arises after organ transplantation, such as diffuse large cell type of lymphoma.	GC	NA	NA
Primary mediastinal B cell lymphoma	A subtype of diffuse B cell large lymphoma located in the mediastinum. Shows similarities to Reed-Sternberg cells. Mostly found in young women.	Thymic B cells	NA	*SOCS1* [40%] (26)
Diffuse large B cell lymphoma	This type of lymphoma is a heterogeneous group typified by large B cells. Immunoblasts and centroblasts show morphological adaptations.	GC or post GC	*BCL6-*various [35%] (27), *BCL2-IgH* [15–30%] (28), *MYC-IgH* or *MYC-IgL* [15%] (29)	*CD95* [10–20%] (30), *ATM* [15%] (31), *TP53* [25%] (32, 33)
Burkitt's lymphoma	An extranodal and fast-growing lymphoma characterized by *MYC-Ig* translocation. Mostly, EBV positive in patients and the sporadic form is present in about 30% of cases.	GC	*MYC-IgH* or *MYC-IgL* [100%] (34, 35)	*TP53* [40%] (36), *RB2* [20–80%] (37)
Splenic MZ lymphoma	Mostly small IgD^+^ lymphoma cells that replace normal follicles and the MZ region. Involves infiltration into the bone marrow and circulation.	Naïve B cells partially differentiated in the MZ	NA	NA
Nodal MZ lymphoma	Present in lymph nodes. The similarity with MZ or monocytoid B cells, with a mostly heterogeneous cytology. Includes plasma cell and lymphocytes range from small to large.	MZ Monocytoid B cells	NA	NA
MALT lymphoma	An extranodal MZ B cell lymphoma that develops in acquired lymphoid structures.	MZ	*API2*–*MALT1* [30%] (38), *BCL10*–*IgH* [5%] (39, 40), *MALT1*–*IgH* [15–20%] (41), *FOXP1*–*IgH* [10%] (42)	*CD95* (5–12, 43–111)
Hairy cell leukemia	Involves the bone marrow and spleen. Few circulating leukemia cells. Cells form hairy projections.	MB	NA	NA
Follicular lymphoma	Resemble GC B cells. Follicular growth pattern. Associated with *BCL2-IgH* translocation.	GC	*BCL2-IgH* [90%] (112)	NA
B cell prolymphocytic leukemia	Chronic B cell malignancy that resembles B cell CLL. More than 50% of cancer cells are prolymphocytes.	MB	NA	NA

*A list of B cell malignancies that have a direct or indirect role in the progression, maintenance, or survival of CLL cells. Numbers in square brackets are the percentages of known cases that carry mutations in this gene. Lymphomas in which the transformation frequency is >5% or the transformation mechanism has not yet been identified are not listed*.

*AIDS, acquired immune deficiency syndrome; EBV, Epstein–Barr virus; Ig, immunoglobulin; MALT, mucosa-associated lymphoid tissue; GC, germinal center; MZ, marginal zone; API2, apoptosis inhibitor 2; ATM, ataxia telangiectasia mutated; B-CLL, B cell chronic lymphocytic leukemia; CCND1, cyclin D1; FGFR3, fibroblast growth factor receptor 3; FOXP1, forkhead box P1; IKBA, inhibitor of nuclear factor-κBα; IKBE, inhibitor of nuclear factor-κBε; MALT1, mucosa-associated lymphoid tissue lymphoma translocation gene 1; PAX5, paired box gene 5; RB2, retinoblastoma-related gene 2; SOCS1, suppressor of cytokine signaling 1; NA, not available*.

CLL also manipulates T cells to gain a survival edge by turning off cytotoxic functionalities of T cells and increased expression of immune checkpoints with abnormal subset distributions. Prior treatment of CLL may also shape the T cells profile (44). CLL cells using their extracellular vesicles (EV) can also modulate T cells in favor of enhanced migration, interaction with tumor cells, and immunological synapse signaling, to avoid immune attack. When analyzed, these EVs contain miR-363 that suppresses the immune modulatory molecule, CD69, and the knockdown of miR-363 altered T cell phenotype (45).

Recently, it has been identified that the lymph nodes harbor the highest number of CLL cells (46), where, the CD4^+^ T cells can induce them to be adhesive toward hyaluronan through CD40L and CD44 interaction and antagonizing their motility (47). The higher number of CD8^+^ T cells as compared to CD4^+^ T cells predispose the CLL patients to a shorter lifespan, and this can be correlated to the immune checkpoint receptor PD-1 expression. CLL patients expressed higher amount of PD-1 due to hypomethylation of its promoter region (48). Further analysis revealed that CLL cell has *CCR6* and *KLRG1* as differentially methylated genes that have known immune regulatory functions. Moreover, a significant correlation was found between T cells and CLL in terms of PD1/PD-L1 interactions when studied in mice model, Eμ-Tcl1 CLL model, and T cells can express a higher level of PD-1 under leukemic cells influence (49).

CLL cells may also interfere with cytotoxic T cell (CTLs) activity and avoid immune surveillance. This can be attributed to the presence of defective linker for activation of T cells (LAT) that is manipulated by B cells. CLL forms a dysfunctional non-lytic immune synapse with CTLs and stimulates CTLs to release non-polarized lytic granules, thus escaping CTL mediated cytotoxicity (50). LAT involvement in clonal expansion and long-term memory was also reported via Ubiquitin Specific Peptidase 9 X-Linked (Usp9X). Ubiquitinated ZAP70 is unable to form functional signalosome with LAT, and Usp9X mediated deubiquitylation of ZAP70 improves signalosome formation in CD4^+^ T cells. Usp9X triggers deubiquitylation under TCR in T cells and similarly activates B cells under BCR for the induction of protein kinase C β (PKCβ) (51). In this way, Usp9X functions to sustain adaptive immune response. The restoration of CTL functions using a combination of GM-CSF and IL-4 (termed as GIFT-4) has been evaluated. GIFT-4 induces CTL to secrete IFN-γ and causes lysis of autologous CLL. GIFT-4 treatment also up-regulated CD40, CD80, and CD86, various interleukins and STAT5 phosphorylation that can convert CLL cells into immune helper-like cells (52).

The imbalances in T cells ratio are a critical proponent for CLL with supporting evidence that the expansion of CD8^+^ T cells in CLL possibly related to a CLL-specific adaptive immune response (53). These imbalances are perpetuated through CD4^+^ forkhead box P3 (FoxP3^+^) regulatory T cells (Tregs) and myeloid-derived suppressor cells (MDSCs) (54, 55). Moreover, it has been reported in several studies that decrease in the number of IL-17^+^, T-helper 17 (Th17) and CTL numbers are associated with poor prognosis (56), while the expansion of Th17 accompanied by lenalidomide might have a defensive role (57). However, the induction of Th17 cells and related cytokines may have the possibility to increase the complication of autoimmune cytopenias (56, 58).

## Innate immune system in CLL: macrophage-DC precursors in CLL pathogenesis

The prominent cells in a myeloid lineage that are crucial for tumor response include macrophages, monocytes, and DCs. Monocytes are acquired from ancestor cells and differentiate either into macrophages or DCs at marginal tissue sites (6). At the beginning of an adaptive immune response, stimulated DCs migrate to secondary lymphoid organs and present the antigen to other antigen presenting cells (59, 60), whereas, after activation, macrophages have tissue-specific functions and remain in the peripheral tissues (61).

Macrophages are usually considered phagocytes of cancer cells and disease-causing particles and they can acquire M1 (antitumor) or M2 (protumor) phenotypes depending on various stimuli and microenvironment (62). Normally, microbial components or IFNγ activates M1 macrophages, which activate T cells, while, the M2 phenotype is triggered by IL-4 and -13 and is involved in controlling the disease response (63), hence macrophage penetration in tumors can be either positive or negative, depending on the tumor type, which suggests the dual role of macrophages (64).

The development of a tumor niche occurs during neoplastic conversion when B cell factors attract circulating monocytes that differentiate into macrophages, which express CD163, CD206, CD14^+^, and CD68 and show an M2-like functionality (64). The differentiated macrophages are functionally indistinguishable from NLCs, rendering TAMs and NLCs functionally equivalent in CLL tissues (65–67). CD14^+^ monocytes from a healthy donor cultured *in vitro* can be transformed into NLCs with CD19^+^ in the presence of CLL B cells; however, normal B cells lack this ability to transform. Differentiation into NLCs promotes the survival of CLL cells via cytokine production. In contrast, healthy B cells are unable to induce an NLC phenotype in monocytes and do not support CLL cell survival (68). Thus, factors that increase the survival of CLL cells are released with the differentiation of NLCs. Moreover, NLCs are blood monocyte-derived cells that secret CXCL12 and 13, and that protect CLL cells from spontaneous apoptosis or drug-induced apoptosis in response to CXCL12, B cell-activating factor (BAFF), a proliferation-inducing ligand (APRIL), CD31, plexin-B1, and activation of the BCR signaling cascade (69–72). NLCs are known as TAMs and LAMs (lymphoma-associated macrophages) in other B cells malignancies; however, the signaling mechanism and roles are similar.

The essential role of chemokines and cytokines in stimulating the symbiotic relationship is supported by monocytes, TAMs/NLCs, and tumor cells. CLL cells secrete IL-4, -10, and -13 (73) that endorse M2-like properties in macrophages and stimulate pro-survival responses through the secretion of IL-8 (74), CCL2, CXCL2, 12 (75, 76), and insulin-like growth factor-1 (IGF-1) (77). Because of the sustained selection of circulating monocytes, the tissue niche is expanded and is rich in chemokines, such as CCL2, 3, and 4 and CXCL12, 13, 19, and 20 (72, 78). Consequently, TAMs/NLCs are promoted by leukemic cells and anti-tumor immunity is suppressed in CLL microenvironments. The properties of TAMs can be modulated by the phosphoinositol-3 kinase/mammalian target of rapamycin (PI3K/mTOR) pathway (79, 80), and they have been shown to suppress T cell-mediated antitumor responses through the PI3Kγ signaling pathway (81, 82). Moreover, gene expression profiling (GEP) of CLL-associated monocytes revealed aberrantly high PD-L1 expression and secretion of multiple inflammatory and immunosuppressive cytokines like IL-10, TNF-α, and CXCL9 that also contribute to worsening the situation.

The functional and clinical importance of DC is long been known, with the functional alteration of cytokine profile in CLL (83), these cells also showed phenotypically immature population with the absence of maturing antigen CD83 and CD80, reduced expression of IL-12 and unable to activate type 1 T cell response (84). Further, unable to induce T cell response can also be coupled to SOCS5 that negatively regulates STAT6, a downstream mediator of the IL-4Rα receptor. In CLL, overexpression of IL-4Rα is due to overactivation of STAT3 and to regulate IL-4Rα activation, SOCS5 comes into play. This decoupling reduced the pro-inflammatory cytokines from DCs and hinders its maturation (85). In another study, the involvement of CXCR5 has been studied in Eμ-Tcl1 CLL model and it has been concluded that CXCR5-deficient cells showed reduced leukemic transformation, and in this activity, follicular DCs play a critical role (86). The disruption of this link may offer a potential therapeutic window.

DC vaccines hold therapeutic potential, and when apo-DC based vaccine along with GM-CSF, lenalidomide, and cyclophosphamide has been evaluated, it triggers a T cell response against tumor cells, however, it showed toxicity that warrants careful administration of this combination (87). The lower induction of TH1 cytokine profile from DC vaccines is an impediment to their clinical efficacy. Recently, it has been reported that α-type-1 polarized DCs (αDC1s) have shown to produce superior TH1 cytokine profile, with an equivalent number of CD70 expression. These αDC1s can be an alternative to prostaglandin-mediated DC maturation for better CLL treatment (88).

## Signaling pathways in CLL

**Figure 2:**
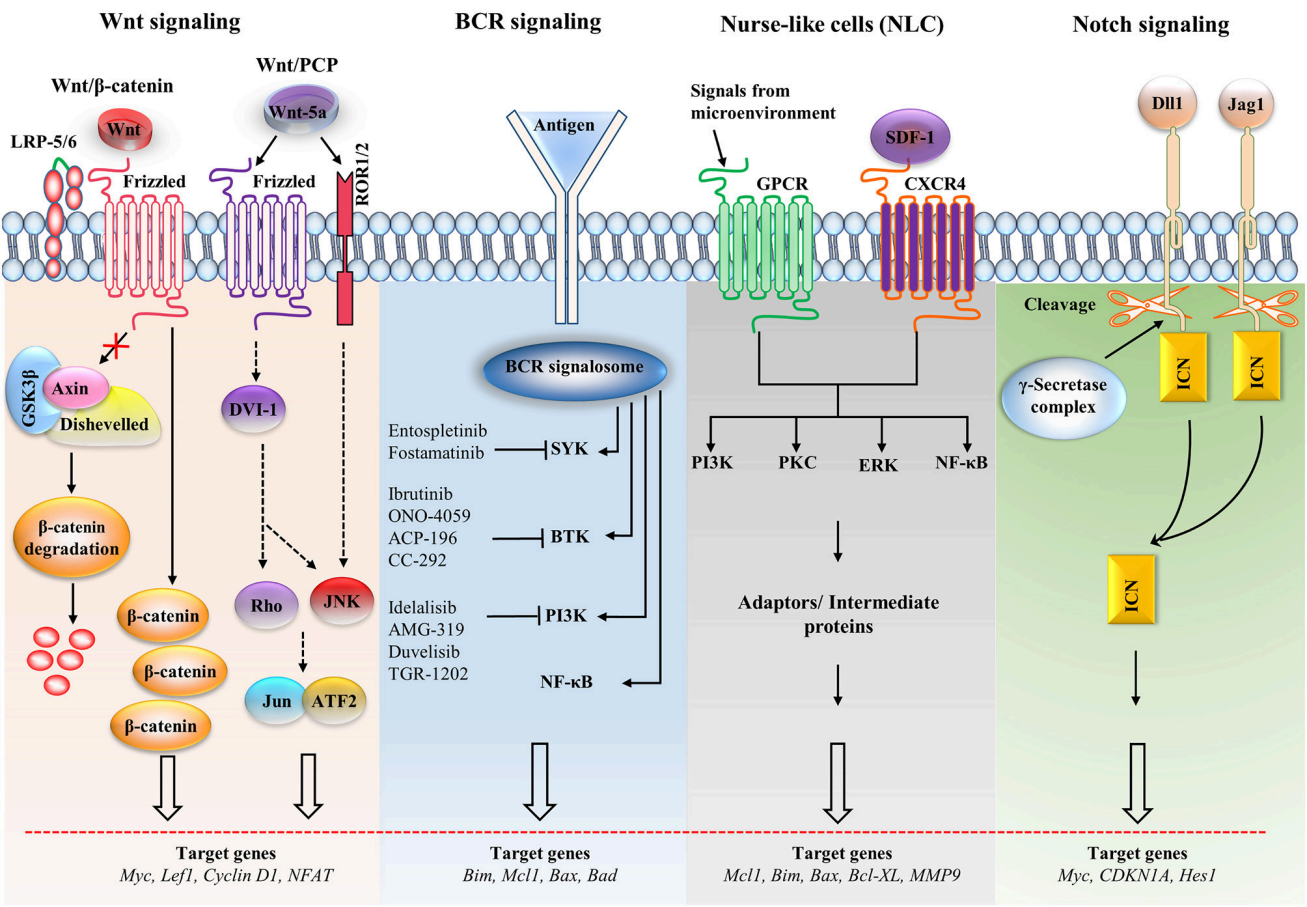
Major signaling pathways and therapeutic targets in CLL cells. The Wnt/β-catenin signaling pathway is activated by the Wnt ligand attached to Frizzled and LRP-5/6 receptors that activate disheveled (dsh), resulting in the inactivation of the destruction complex. This inactivation causes the accumulation of β-catenin, which enters the nucleus. The nuclear translocation of β-catenin allows it to interact with the transcription factor TCF/LEF and induces the transcription of the target genes. The non-canonical Wnt pathway [Wnt/planar cell polarity (PCP)] is triggered by Wnt-5a that enhances the heterodimerization of receptor tyrosine kinase-like orphan receptor 1 (ROR1) and ROR2. Receptor binding leads to the formation of the ligand-receptor complex and downstream activation of the dsh protein (DVI-l), Rho GTPases and c-Jun N-terminal kinase (JNK), which together regulate tissue polarity and cell motility, and has also been implicated in organogenesis and cancer metastasis. B cell receptor (BCR) signaling causes a signalosome containing spleen tyrosine kinase (SYK), Bruton's tyrosine kinase (BTK), phosphatidylinositol 3-kinase (PI3K), and adaptor proteins to form, which activates downstream pathways. Small molecule inhibitors are shown in the BCR pathway. In nurse-like cells (NLCs), the interaction of chemokines with receptors generates microenvironmental signaling. NLCs regulates several signaling pathways, including the protein kinase C (PKC), PI3K, extracellular regulated kinase (ERK), and nuclear factor-κB (NF-κB) pathways, which are similar to the BCR pathway. In the Notch signaling pathway, the ligands [Dll1, Jagged-1 (JAG1)] bind with the receptor and cause the intracellular Notch domain (ICN) to be cleaved by the g-secretase complex. The cleaved domain then translocates to the nucleus, forms complex with other proteins and activates target genes. *BAD*, Bcl2-associated agonist of cell death; *Bcl-XL*, B-cell lymphoma-extra-large; *CDKN1A*, cyclin-dependent kinase inhibitor 1A; *GSK3*β, glycogen synthase kinase-3β; *GPCR*, G-protein coupled receptor; *LEF1*, Lymphoid enhancer-binding factor 1; *LRP*, lipoprotein receptor-related protein; *MMP*, matrix metalloproteinase; *SDF-1*, stromal cell-derived factor 1.

In CLL, numerous pathways play essential roles in responding to external stimuli, making identification of the most significant pathway a challenge. Several pathways are involved in the proliferation and survival of CLL cells, including the mitogen-activated protein kinase/extracellular regulated kinase (MAPK/ERK), nuclear factor κB (NF-κB), Notch, Wnt, phosphatidylinositol-3-kinase/AKT (PI3K/AKT), and Janus kinase/signal transducers and activators of transcription (JAK/STAT) pathways (89). The stimulation of signaling pathways, specifically MAPK/ERK, NF-κB, and PI3K/AKT, in a tissue microenvironment is dependent upon the composition of the microenvironment. This can differ among different tissues, triggering specific signaling pathways (Figure 2) (90).

The BCR signaling pathway is the most critical pathogenic factor, and it has long been considered a valuable target in CLL. In various studies, BTK (kinase factor of BCR) inhibitor has been evaluated with other chemotherapeutic agents to reduce the pathophysiology of CLL (72). Continuous activation of the BCR leads to the apoptosis-resistant CLL cells, and overexpression of the antiapoptotic proteins XIAP, MCL-1, and BCL-XL (91). Moreover, studies involving ibrutinib and CAL101 (PI3K inhibitor) have shown promising results in the treatment of CLL (92). Furthermore, evaluation of a highly selective oral AKT inhibitor, MK2206, indicated that this compound selectively inhibits BCR-induced cytokines, activates other lymphocytes, and in synergy with bendamustine, induces apoptosis in CLL cells (93).

The MAPK/ERK pathway conveys pro-survival signals in cancerous cells and is mainly activated by various growth factors, and CCL19, 21 and CXCL12, 13 stimulation (94, 95). The basic components of this pathway include one small G protein (Ras) accompanied by three other kinases (RAF, MEK, ERK) that, upon activation, leads to the translocation of the ERK to nucleus and activation of target genes (96). The entry of CLL cells into the S-phase of the cell cycle and expression of MYC are essential activities of MEK1/2, and MEK2 also upregulates antiapoptotic protein, XIAP (91, 95). MYC induces the cell cycle component cyclin D2 and downregulates p27, a cell cycle inhibitor (97, 98).

The NF-κB pathway generally promotes proliferation and is downstream of many cell surface receptors, including Toll-like receptors (TLRs). These receptors can be triggered through a multitude of signals, leading to the activation of NF-κB, which induces the transcription of CCL5, 9, 17, 20, and 22, IFNγ, IL-2, 6, 8, 9, and 10, MIPs, CCRs, CXCRs, TLR 2 and 9, and various other early response genes, transcription factors, and regulators (99). It also cross-talks with various other pathways; for instance, stimulation of NF-κB upregulates *BFL-1* and *B cell lymphoma-extra-large* (*BCL-XL*) anti-apoptotic genes, which inhibit the apoptosis of CLL cells via PI3K/AKT signaling. Further, IL-4 and soluble CD40-ligand (sCD40L) were found to be most effective in preventing CLL apoptosis by triggering NF-κB (100). NF-κB also modulates the redox state of cells, and, when production of reactive oxygen species is blocked using IT901, tumor cells become susceptible to apoptosis (101).

Notch signaling pathway plays a role in embryonic development, cell fate determination, and neural differentiation. There are four different Notch receptors: Notch1, Notch2, Notch3, and Notch4. A mutation in the Notch1 receptor has been confirmed in 10–15% of CLL patients (102). The initial events in this pathway involve the interaction of Jagged and Delta-like ligand with the Notch receptor, induces its cleavage by γ-secretases, and the cleaved Notch1 receptor forms a complex with other factors that stimulate the transcription of target genes, including HES1 and Myc. In CLL, Notch1 and Notch2 and their ligands are constitutively expressed, which allow cells to resist apoptosis, upregulates NF-κB activity, and induces expression of XIAP and cIAP2 (cellular inhibitor of apoptosis protein 2). However, inhibition of Notch signaling by γ-secretase inhibitor and specific siRNAs promotes the death of CLL cells (103). Furthermore, Notch signaling is also involved in the induction of MCL-1 and the promotion of eukaryotic translation initiation factor 4E activity that leads to CLL survival (104). Inhibition of the Notch signaling pathway may also induce the expression of Kruppel-like factor 4, which was suppressed due to aberrant methylation (105).

The Wnt signaling pathway plays an important role in cell development, differentiation and oncogenesis. This pathway shows a high level of Wnt and Frizzled expression, and downregulation of antagonist genes, including *WIF1* and secreted Frizzled-related members (*SFRP*). Wnt activation inhibits GSK3β mediated β-catenin phosphorylation, then the dephosphorylated β-catenin enters the nucleus and binds with lymphoid enhancing factor (LEF) to stimulate the transcription of Wnt target genes, such as *LEF, CyclinD1, Myc*, matrix metalloproteinases, and *cyclooxygenase-2* (106–108). During CLL, overexpression of the Wnt pathway and enhanced translocation of β-catenin to the nucleus occurs in the absence of E-cadherin (106). In a recent report, ibrutinib, a classical BTK inhibitor, was used to target metadherin, that not only inhibited metadherin, but also LEF1 and β-catenin (109). Other than canonical Wnt, recently, Wnt5a was found to act as a regulator of ROR1, a receptor in the non-canonical Wnt/planar cell polarity (PCP) pathway, which may promote CLL cell survival and subvert apoptotic signals (110).

PI3K/AKT signaling pathway is a central mediator of cancerous cells and their microenvironment and transmits signals from CXCR4 and CD40 (91). The principal regulator of this pathway is phosphatase and tensin homolog (PTEN) (113), along with other transcription factors, such as FOXO. Upon activation of PTEN, FOXO proteins are translocated to the nucleus and induce p27, which arrests the cell cycle. Activation of the PI3K/AKT pathway by microRNA-22 induces the proliferation of CLL cells, which can be reversed by inhibiting the PI3K-Δγ signaling pathway using IPI-145 (114, 115). The role of the PI3K/AKT pathway in CLL proliferation can be attributed to a chemotactic response mediated by CXCL12 and 13 and CCL19 and 21 (69, 116) and assists in the survival of cancerous cells in response to various exterior stimuli conveyed through CD40L (117), BCR (91), CCL19 and 21, vascular cell adhesion molecule 1, and anti-apoptotic proteins, such as BCL-2 and MCL-1 (90). Moreover, synergy was observed in PI3K/AKT and Hedgehog (HH)/GLI pathway, when both these targeted simultaneously, a synergistic therapeutic effect is observed in CLL, which suggests a combinatorial therapy (118).

The JAK/STAT signaling pathway is another pathway crucial that mediates signals from cytokines, which are soluble messengers produced by various cells. The JAK/STAT pathway provides a direct link from the cell surface to the nucleus. The binding of the cytokine to its cognate receptor triggers this pathway that involves phosphorylation among JAK and STAT molecules. Later, STAT molecules translocate to the nucleus and bind with DNA as either hetero- or homodimers. There are multiple JAK and STAT molecules that combine in different ways to induce diverse transcriptional profiles (119). In CLL, inhibition of the JAK2/STAT3 pathway culminates in the apoptosis of cancerous cells. The dual inhibitor cerdulatinib, which targets both spleen tyrosine kinase (SYK, a BCR component) and JAK kinases, potently inhibits tumor growth and induces apoptosis in CLL cells at clinically feasible drug concentrations (120).

## Innate vs. adaptive immune system: an interplay of myeloid and lymphoid cells in CLL

The interaction between lymphocytes and myeloid cells is crucial for immune response and any anomaly may prone the individual at risk of developing diseases. B cells present in the MZ, a region between red and white pulp of spleen and enriched with macrophages, are an essential link between innate and adaptive immunity (121, 122). Recently, acquisition of antigen through BCR and then transfer to macrophages through direct contact has been reported (7) that signifies B cells involvement in macrophage-mediated CD4^+^ T cells activation. Further, B cell development into memory or plasma cells needs to encounter an antigen, which can either be diffusion-controlled or be presented by macrophages, DCs, or FDC (123).

**Figure 3:**
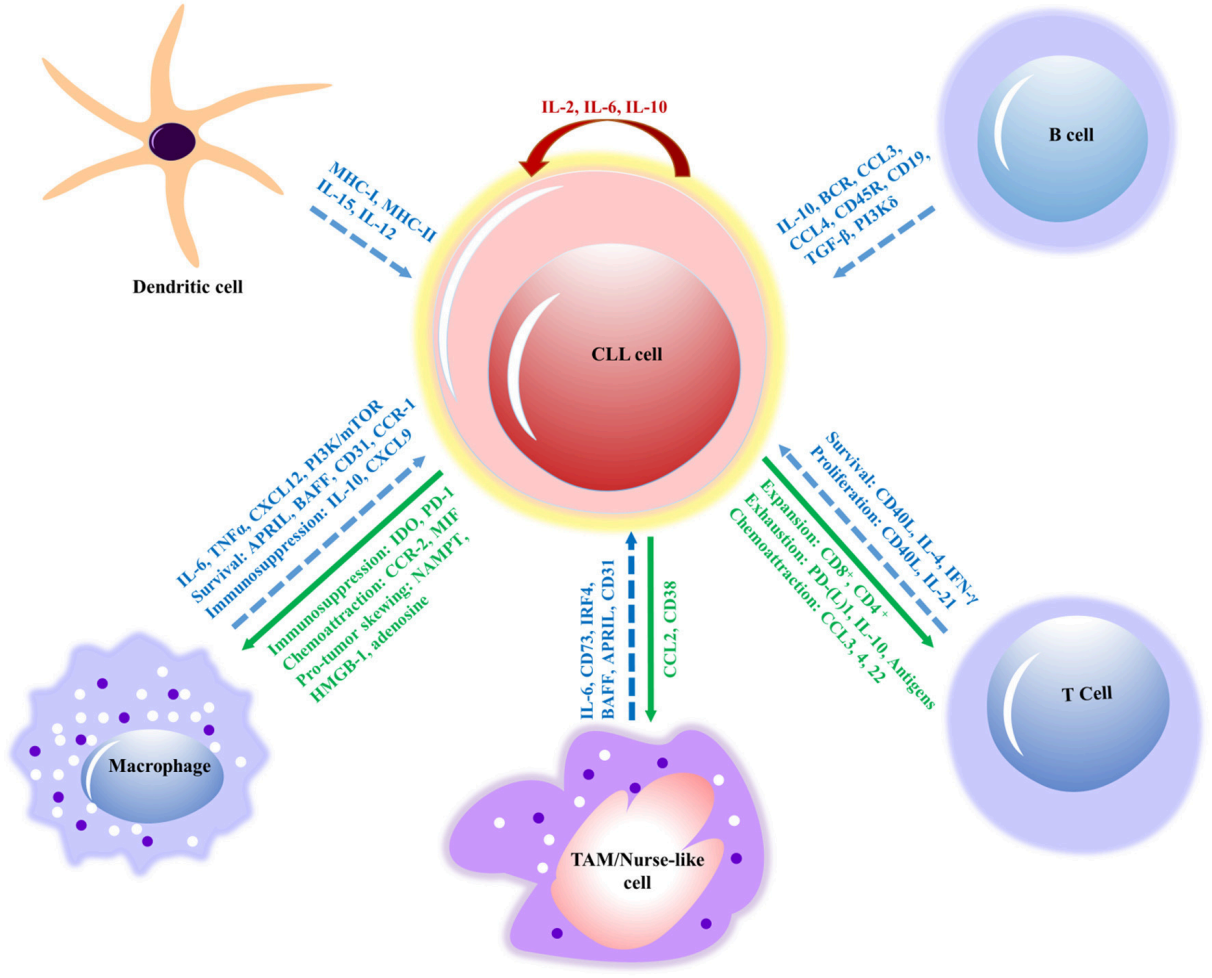
Interactions between CLL cells and bystander cells that subsidize the formation of a tumor-supportive microenvironment. The molecular interactions of innate immune cells (macrophages, dendritic and nurse-like cells) and adaptive immune cells (B and T cells) have relevant effects, such as survival, immunosuppression, proliferation and signaling molecules (in blue) involved in the interactions of CLL cell. The response of CLL cells includes exhaustion of T cells, expansion, chemoattraction, immunosuppression, and immune evasion (in green) on innate and adaptive immune cells. However, IL-2 and IL-10 (in red) contribute to the autocrine self-renewal and survival of CLL cells. *APRIL*, a proliferation-inducing ligand; *BAFF*, B cell activating factor; *BCR*, B cell receptor; *CCL*, chemokine (C-C motif) ligand; *CD*, cluster of differentiation; *CLL*, chronic lymphocytic leukemia; *CSF1R*, colony-stimulating factor-1 receptor; *CSF1*, colony-stimulating factor-1; *CXCL*, chemokine (C-X-C motif) ligand; (TGF-β), Transforming growth factor beta; *NAMPT*, extracellular nicotinamide phosphoribosyltransferase; *HMGB1*, High mobility group box 1; *IL*, interleukin; *MIF*, mini zinc finger; *PI3K/mTOR*, phosphatidylinositol-3-kinase/mammalian target of rapamycin; *TAM*, tumor-associated macrophage; *MHC*, major histocompatibility complex; *TNF*, tumor necrosis factor; *CCR*, chemokine receptor; *IDO*, indoleamine 2,3-dioxygenase; *PD-1*, program cell death; *IRF4*, interferon regulatory factor; *IFN*, interferon.

B cells and macrophages show bidirectional interactions via different soluble factors. Macrophages produce APRIL and BAFF in the presence of costimulatory signals, such as IL-6, TGFβ, IL-10, and TLR ligands (124, 125). In proliferating B cells, BAFF is significantly involved in signals exchanged between macrophages and B cells. Additionally, B lymphocyte stimulator (BLyS), a TNF family member, also induces the B cell growth. The B cell tropism of BLyS is consistent with its receptor expression on B-lineage cells. The biological profile of BLyS suggests that it is involved in monocyte-driven B cell activation (125, 126). IL-10 involved in the initial development of B1 cells, and IL-6 contributing to later B cell development (127, 128). Moreover, the accumulation of B cells in response to IL-10 can suppress murine macrophage functions *in vitro* (129) and transform macrophages into cells with a pro-tumor phenotype both *in vivo* and *in vitro* (Figure 3) (130).

GEPs indicate complex interactions between macrophages and malignant B cells in the microenvironments that induce cellular changes. This analysis has shown that CLL cells are activated differently by bone marrow-derived stromal cells (BMSCs) and NLCs (72, 131). In particular, the expression pattern induced by BMSCs shows a characteristic upregulation of T cell leukemia/lymphoma 1 (TCL1), a lymphoid proto-oncogene, with a concomitant decrease in TCL1-interacting FOS proto-oncogene and Jun proto-oncogene (FOS/JUN) (131). However, an NLC-induced GEP pattern in leukemic cells showed the expression of genes in the NF-κB and BCR signaling pathways (72), which was astonishingly similar to the expression pattern of CLL cells that were extracted from a patient's lymph nodes (132). Additionally, BMSCs differentially induce many vital genes (e.g., TNF receptor superfamily member 17 (TNFRSF17), pre-B lymphocyte 3, TNFSF10), but their precise functions in the CLL microenvironment remain to be explored. MDSCs have also been widely studied in the context of immune and T cells suppression in malignant diseases. MDSC (CD14^+^/HLA-DR^−/−^) was increased in CLL that suppress T cell activation and caused suppressive Tregs activation and this can be attributed to increased indoleamine 2,3-dioxygenase activity in T cells (55).

The interaction of leukemic cells with bone marrow cells is perpetuated through adhesion molecules and chemokines (133), and cell-to-cell contact via bidirectional interplay as studied by Hacken and Burger (134). The CXCR5 expression on leukemic cells controls the approach to FDCs, which provide the proliferative stimuli and expression of myeloid cell leukemia-1 (MCL-1) antiapoptotic protein (86, 135). The stimulation or suppression of the development of CLL clones by T cells have been reported, suggesting that T cells are unable to form efficient immunological synapses alone (136). On the other hand, colocalization with CLL cells indicates that they may induce the development of leukemic clones (133). There are several T cell-attracting chemokines, such as CCL3 and 4, which are secreted at high levels by CLL cells in response to TNF-α, IFNα, and IL-2 that protect cancerous cells from apoptosis (72, 134).

Human signaling lymphocytic activation molecule family 1 (SLAMF1/CD150) receptor is expressed by T cells, B cells, macrophages, and DC, where it acts as a co-stimulatory through self-interactions on the hematopoietic cell surface, and plays critical roles for various T and B cells functions. In a subset of CLL patients, SLAMF1 was found to be downregulated that warranted the shorter life spans (137). Moreover, SLAMF1^−/−^ positively affects chemotactic response to CXCL12 by overexpression of CD38, CD44, and CXCR4, and its activation through antibody can facilitate the autophagic flux. SLAMF1 along with other receptors, particularly, CD180, influences the leukemic cells pathobiology. This signaling crosstalk causes the inhibition of AKT and MAPK pathways and results in reduced phosphorylation of ERK1/2, AKT, ribosomal S6 kinase, c-Jun, and other vital intermediate kinases (138, 139). Thus, various studies revealed that SLAMF1 plays a critical role in CLL pathogenesis and restoring the expression of SLAMF1 would be a great therapeutic target for CLL.

## Therapeutic implications

CCL is either resistant to chemotherapy or the outcome is very poor. Consequently, various studies are underway to develop new therapies that either inhibit intracellular signaling or block extracellular signals using small molecules, antibodies, kinase inhibitors, immunomodulators, and antagonists (Table 2) (90).

**Table 2:** Current therapies that are under investigation for use in chronic lymphocytic leukemia.

**Class**	**Agent**	**Target**	**Phase of study**	**Status**	**Side effects**	**Reference/ clinicaltrials.gov**
Small molecules/ antibodies	Obinutuzumab	CD20	III	Completed	Transfusion-related reactions	NCT01680991
	BI 836826 (moAb 37.1)	CD37	I	Active, not recruiting	Liver enzymes	NCT01296932
	Otlertuzumab	CD37	I/II	NA	Infusion reaction, nausea, fatigue, diarrhea	(140)
	Blinatumomab	CD19	I	Recruiting	Cytokine release syndrome	NCT02568553
	Rituximab	CD20	II	Terminated	Nausea, heartburn, joint pain, diarrhea	NCT01625741
Kinase inhibitors	Idelalisib	PI3Kδ	III	Active, not recruiting	Pneumonitis, diarrhea	NCT01539291
	AMG319	PI3Kδ	I	Completed	Diarrhea	NCT01300026
	IPI-145	PI3Kγδ	I/II	Active, not recruiting	Pneumonitis, neutropenia	NCT02158091
	Ibrutinib	BTK	III	Active, not recruiting	Diarrhea	NCT02801578
	AVL-292	BTK	I	Completed	Cytopenia	NCT01351935
	ONO-4059	BTK	I	Completed	NA	NCT01659255
	Dasatinib	BCR-ABL, Src	II	Completed	Pleural effusion	NCT00438854
	Fostamatinib	SYK	I/II	NA	Fatigue, diarrhea, nausea	(140)
	GS-9973	SYK	I	Completed	NA	NCT01841489
	CC115	mTOR, DNA-PK	I	Active, not recruiting	Hyperglycemia	NCT01353625
	PRT-2070	SYK	I (pending)	NA	NA	NA
BCL-2 antagonists	ABT-199	BCL-2	II	Active, not recruiting	Neutropenia, tumor lysis syndrome	NCT02141282
Immunomodulators	Lenalidomide	Multiple	III	Terminated	Myelotoxicity, tumor flare, immunosuppression, thromboembolic events	NCT00751296
CARTs	CTL019	CD19	II	Recruiting	B cell depletion, cytokine release syndrome	NCT02228096

*CARTs, chimeric antigen receptor transduced T cells; moAb, monoclonal antibody; PI3K, phosphoinositide 3-kinase; BTK, Bruton tyrosine kinase; mTOR, mammalian target of rapamycin; DNA-PK, DNA protein kinase; NA, not applicable*.

The role of colony stimulating factor 1 (CSF1/CSF1R) in regulating the survival and differentiation of macrophages is well-recognized, and blocking signaling through this pathway may have therapeutic significance (141, 142). The activity of CSF1R is blocked by the inhibitor pacritinib, which reduces NLCs and limits the progress of CLL (143). Importantly, a novel treatment, anti-CSF1R monoclonal antibody, is under clinical assessment in solid tumors (144). It has also been observed that CSF1R blockade targets TAMs and reprograms the microenvironment to an antileukemic phenotype. Targeting macrophages not only increases the death of CLL cells but also that of CD20^+^ leukemic cells (145). In a recent clinical trial, patients were given ibrutinib alone and in combination with anti-CD20 monoclonal antibodies (moAbs). The study revealed that ibrutinib had both positive and negative effects, as ibrutinib consistently downregulated CD20, rendering cells less susceptible to moAb. However, ibrutinib can impair trogocytosis, a major contributor to antigen loss and tumor escape during moAb therapy (146). Moreover, macrophage targeting through CSFR1 blockade can also increase CD20^+^ leukemic cells, and targeting of macrophages by either CSF1R signaling blockade or clodronate-induced macrophage killing can significantly inhibit established leukemia. The removal of macrophages facilitates TNF-mediated leukemic cell death and modifies the microenvironment to induce an antitumor response. By inhibiting CSF1R, it reduces the CLL cell population and increases CD20^+^ CLL cells that can be co-targeted along with TAM to achieve superior results (145).

Recently, a scavenger receptor, MARCO, was identified on TAMs that is immunosuppressive and is present in the tumor microenvironment. This receptor is specific for a subtype of TAMs and has been successfully targeted through moAbs in various solid tumors, where it has antitumor effects (80). The antitumor effects obtained by targeting this receptor is proposed to be mediated by the Fc-receptor FcγRIIB. Although it has not been evaluated in CLL, it presents a potential target for therapy. Trabectedin is used for the treatment of soft tissue sarcomas, where it reduces TAMs (147). It is cytotoxic for human monocytes and inhibits the production of IL-6 and CCL2; in addition, it may shape the microenvironment of chronic B lymphoid malignancies (147). CD47 is another target molecule that needs to be explored in CLL as a potential therapy (148). Lenalidomide is an immunomodulatory drug used for the treatment of CLL, (149) and showed rare cytotoxicity in malignant cells, but apparently, it acts on tumor-supporting microenvironments and affects the CLL myeloid compartment (150).

Valproic acid (VPA) is a histone deacetylase inhibitor (HDAC/HDI), which has long been used as an antiepileptic drug. VPA has also been evaluated in hematological malignancies for the apoptosis induction of cancerous cells, and has been clinically tested to cause differentiation in carcinoma cells, acute myeloid leukemia cells from patients, and transformed hematopoietic progenitor cells. It has also reduced the tumor growth in animal models (151, 152). VPA perpetuates its effects by up-regulating cyclin-dependent kinase inhibitor 1A, BCLA1, and p53, whereas proto-oncogene (c-Myc, BCL2, BCL-XL, Ataxia-Telangiectasia Mutated, protein kinase B) were downregulated, thereby augmenting mTOR inhibitor to induce autophagic cell death in several kinds of lymphomas including Burkitt leukemia/lymphoma, cutaneous T cell lymphoma and CLL (153, 154). Furthermore, VPA alone or in combination with other drugs, such as fludarabine, flavopiridol, cladribine, lenalidomide, and bortezomib is highly effective in inducing cell death in CLL cells and could be considered for therapeutic intervention (155, 156).

Immune checkpoint inhibitors (ICI) are gaining popularity for their therapeutic potential as these ICIs block the interaction of cytotoxic T lymphocyte-associated 4 (CTLA4) and PD-1 to their cognate ligands, CD80/CD86 and PD-1 ligand (PD-L1), respectively. These interactions are critical for cancer cell survival including CLL in the tumor microenvironment (157). Various ligands and antibodies have been developed to target this particular interaction. Recently, chimeric antigen receptor (CAR) T cell therapy is being studied that targets B cell-specific antigen in CLL, CD19 (anti-CD19 CART [CTL019]), and observed a promising response in clinical trials for CLL patients (158). In future, CAR-T cells combining with other therapeutic agents could be the treatment of choice for various malignancies.

## Future prospect and conclusion

B cells and macrophages are crucial components of the immune system. These cells interact and influence each other at various levels, beginning in development and later inactivation and immune responses. There are several links between these cells that are either protumoral or antitumoral. In this review, we provided a sketch of B cells and macrophages and discussed their links. The molecular interactions between macrophages and B cells have been characterized in detail in CLL. There are various mutations that cause CLL, affecting different pathways as either causative agents or enhancers of CLL pathogenicity, as well as, a plethora of signals from the microenvironment and the interplay of various other cells that play roles in the progression of CLL. All these aspects complicate the mechanistic elucidative studies, hinder the formulating and devising therapeutic strategies, and complicate the outcome of already implemented therapeutic regimens.

Several pathways are involved in CLL. The real problem arises when these pathways interact with other pathways to increase the overall intensity or exaggerate the outcome. These interactions are surprisingly difficult to manage in cancer cases, and particularly in CLL. Moreover, CLL is a neoplasm of immune-related cells, adding an extra layer of complexity, because any immunotherapy approach that activates immune cells can also activate tumor cells.

Consequently, we assessed that single molecule therapy is often inadequate for CLL as these offer a wide range of interactions that simultaneously propagate the disease, suppress the immune system, and resist the therapeutic intervention. Therefore, we emphasized on the common targets such as, PD-1, BTK, CSF1R, and CD20 that were in one cell influence the phenotype of other cells rendering the possibility of co-targeting multiple cells simultaneously in order to achieve superior results.

## Author contributions

MH and MA conceptualized and drafted the manuscript. MH created the tables. MH and SC created the figures. SC critically analyzed and coordinated the project. All authors read and approved the final manuscript.

### Conflict of interest statement

The authors declare that the research was conducted in the absence of any commercial or financial relationships that could be construed as a potential conflict of interest.

## Abbreviations

ADP: antibody dependent phagocytosis
Ag: antigen
AIDS: acquired immune deficiency syndrome
APC: antigen-presenting cells
API2: apoptosis inhibitor 2
APRIL: A proliferation inducing ligand
ATM: ataxia telangiectasia mutated
BAFF: B cell activating factor
B-CLL: B cell chronic lymphocytic leukemia
BCR: B cell receptor
BM: bone marrow
BTK: bruton tyrosine kinase
CARTs: chimeric antigen receptor transduced T cells
CCL3: C-C motif chemokine ligand 3
CCL4: C-C motif chemokine ligand 4
CCND1: cyclin D1
CD: cluster of differentiation
CDR3: complementarity determining region 3
CEBPα: CCAAT/enhancer-binding protein α
CEBPβ: CCAAT/enhancer-binding protein β
CLL: chronic lymphocytic leukemia
COX-2: cyclooxygenase-2
CSF1R: colony-stimulating factor 1 receptor
CXCL13: chemokine (C-X-C motif) ligand 13
CXCR5: C-X-C chemokine receptor type 5
DC: dendritic cells
EBV: Epstein-Barr virus
Fcγ R: Fc γ receptor
FDCs: follicular dendritic cells
FGFR3: fibroblast growth factor receptor 3
FOXP1: forkhead box P1
GC: germinal center
GSK3β: glycogen synthase kinase 3β
IFNα: interferon α
Ig: immunoglobulin
IGF-1: insulin-like growth factor-1
IKBA: inhibitor of nuclear factor-κBα
IKBE: inhibitor of nuclear factor-κBε
IL-2: interleukin 2
IL-6: interleukin 6
LEF: lymphoid enhancing factor
LNs: lymph nodes
MALT: mucosa-associated lymphoid tissue
MAPK: mitogen-activated protein kinase
MB: memory B cell
MCL-1: myeloid cell leukemia
MMPs: matrix metalloproteinases
moAbs: monoclonal antibodies
mTOR: mechanistic (mammalian) target of rapamycin
MZ: marginal zone
NF-κB: nuclear factor-κB
NK: natural killer cell
NLCs: nurse-like cells
*PAX5*: paired box gene 5
pHSCs: pluripotent hematopoietic stem cells
PI3Kγ: phosphoinositide 3-kinase
PKC: protein kinase C
PU.1: purine rich sequence box
*RB2*: retinoblastoma-related gene 2
SOCS1: suppressor of cytokine signaling 1
SYK: spleen tyrosine kinase
TAMs: tumor-associated macrophages
TNF-α: tumor necrosis factor-α
WIF1: WNT inhibitory factor 1.
